# Optimisation of the Photonic Efficiency of TiO_2_ Decorated on MWCNTs for Methylene Blue Photodegradation

**DOI:** 10.1371/journal.pone.0125511

**Published:** 2015-05-18

**Authors:** Nura Abdullahi, Elias Saion, Abdul Halim Shaari, Naif Mohammed Al-Hada, Aysar Keiteb

**Affiliations:** Department of Physics, Faculty of Science, University Putra Malaysia, Serdang, Selangor, Malaysia; University of Aveiro, PORTUGAL

## Abstract

MWCNTs/TiO_2_ nanocomposite was prepared by oxidising MWCNT in H_2_SO_4_/HNO_3_ then decorating it with TiO_2_-p25 nanopowder. The composites were characterised using XRD, TEM, FT-IR PL and UV−vis spectroscopy. The TEM images have shown TiO_2_ nanoparticles immobilised onto the sidewalls of the MWCNTs. The UV-vis spectrum confirms that the nanocomposites can significantly absorb more light in the visible regions compared with the commercial TiO_2_ (P25). The catalytic activity of these nanocomposites was determined by photooxidation of MB aqueous solution in the presence of visible light. The MWCNTs/TiO_2_ (1:3) mass ratio showed maximum degradation efficiency. However, its activity was more favourable in alkaline and a neutral pH than an acidic medium.

## Introduction

Intensive efforts have been made by various researchers in the development of photocatalytic materials that will utilise visible light energy and thus resolve the increasing global concerns of environmental protection and environmentally friendly fuel production [1]. Titanium oxide (TiO_2_) is one of such materials that are active for solar energy conversion due to its nontoxicity, low cost, physical, chemical and thermal stability, availability, and unique electronic and optical properties [2, 3]. But, its efficiency is limited because of its large band gap and high rate of electron-hole recombination resulting in low quantum efficiency. To overcome these difficulties and shift the threshold of the light response of TiO_2_ to the visible region, different techniques have been employed. One is the use of chemical methods, such as coupling with secondary semiconductors, photosensitisation of dye, and doping with transition metals (Au, Pd, Pt, Rh, etc.) [4, 5] or nonmetal elements (N, S, I, F, etc.) [6, 7]. The other is to use physical methods, such as introducing microwave or ultrasonic irradiation into TiO_2_ photoreaction systems [8, 9]. These days’ substantial efforts have been directed towards MWCNT/TiO_2_ composite materials in an attempt to exploit the synergetic effects of their integrated intrinsic outstanding properties and thus improve the activity to meet potential challenges for specific applications. However, due to the one dimensional nature of CNTs, charge carriers can travel through nanotubes without scattering leading to ballistic transport that minimises Joule heating thus facilitating carrying very large current densities [10]. These transporting properties of CNTs have been proven to provide a convenient means of directing the flow of photogenerated electrons and holes and increase the lifetime of electron-hole pair generation [11, 12]. The synthesis of the TiO_2_/CNT nanocomposites using plasma-enhanced chemical vapour deposition, sol–gel method, physical mixing technique, and the nanocoating-hydrothermal process has been reported [13–16]. Fan and co-workers reported an improved decolourisation in an azo-dye solution under (UV) light irradiation with an increase in the concentration of (MWCNTs) in a suspension of Degussa P25 [17]. There are a lot of reports on the determination of photocatalytic activity of CNT/TiO_2_ composites using methylene blue (MB) as a sample pollutant under visible light irradiation [15, 18–20]. But there are inadequate reports on the influence of mass ratios, initial dye concentration as well as the influence of the pH of the dye aqueous solution.

In this work, visible-light responsive MWCNTs/TiO_2_ composites were prepared by oxidising MWCNTs with H_2_SO_4_/HNO_3_ and decorating the nanotubes’ surfaces with TiO_2_ nanopowder. The nanocomposites were finally sintered at 300°C. The photocatalytic activities of these nanocomposites were investigated via the degradation of methylene blue (MB) with visible light irradiation. The composites proved to be active photocatalysts within the visible light spectrum, driving efficiently, the photochemical degradation.

## Experimental

Milli-Q (Millipore, Billerica, MA, USA) water was used in all experiments. All other reagents, such as Carbon nanotube multi walled >90% carbon basis, D x L (110–170 nm x 5–9 μm), titanium (IV) oxide nanopowder, 21 nm particle size > = 99.5% trace metal basis, methylene blue (dye content > = 82%), concentrated nitric and sulfuric acids, ethanol 95% were of analytical grades and were all purchased from Sigma-Aldrich, and used without further purification.

### 1. Acid treatment of the MWCNTs

2g of the MWCNTs were suspended in a mixture of concentrated nitric and sulfuric acids in the ratio of (HNO_3_/H_2_SO_4_1:3). The mixture was sonic treated for 3 hours and then placed on a magnetic stirrer at 400 rpm and heated at 50°C for 24 hours. This oxidation reaction introduced Carbonyl, carboxylic and hydroxyl functional groups that served as nucleation and anchor sites for the metal oxide nanoparticles.

The oxidation process was quenched by the gradual addition of the mixture into deionised water, and finally washed several times with deionised water until the pH was 7.0. The MWCNTs were dried in an oven at 100°C and termed Functionalised MWCNTs (fMWCNTs).

### 2. Preparation of the MWCNTs/TiO_2_ composites

For the composite preparation, three MWCNTs/TiO_2_ composites of different TiO_2_ doses were prepared, namely, (1:1), (1:2) and (1:3). The preparations were made by dispersing weighted amounts of MWCNTs and TiO_2_ in ethanol and ultrasonicated for 2 hours; then, they were magnetically stirred at 70°C for 30 min to enable the adhesion of TiO_2_ on the tubes. The solutions were finally dried at 100°C and sintered at 300°C for 4 hours to obtain the MWCNTs/TiO_2_ composites. For comparison, TiO_2_ nanopowder was used as a reference in the photodegradation reaction.

#### Characterisation

The degree of crystallinity of the MWCNT/TiO_2_ nanocomposites was investigated by the X-ray Diffraction spectroscopy (XRD) technique using a Schimadzu diffractometer (Model: XRD 6000) with Cu K (0.154 nm). The Fourier transform infrared (FTIR) spectra of the pristine and acid treated MWCNTs were recorded using the FTIR spectrophotometer (JASCO-460 PLUS, Japan). The morphology of the MWCNTs/TiO_2_ composites were visualised using (JOEL 2010F UHR version electron microscope) (TEM) operating at an accelerating voltage of 200kV. Photoluminescence spectroscopy was performed on a Perkin Elmer LS 55 fluorescence spectrometer with different excitation wavelength at room temperature. The measurement accuracy of the recorded wavelengths was about ± 0.5 nm. The optical properties of the nanocomposites as well as their photocatalytic activities were studied using the Ultra violet visible spectrometer (UV-vis.) model (Shimadz-UV1650PC).

### 3. Photocatalysis experiment

The photocatalytic activity of the composites as well as the TiO_2_ nanopowder was evaluated by the photodegradation of MB. Taking 0.5 L of 10 ppm MB solution in a cylindrical pyrex reactor containing 0.1g of the catalyst at pH 7 [21]. Adsorption and desorption equilibrium was attained by agitating the suspensions with a magnetic stirrer in a dark condition for 30 mins and an aliquot was taken before the suspensions were exposed to visible light irradiation. The solutions were irradiated with a 500 watt halogen lamp as the source of the visible light without a filter (emission range 400–800nm) and air bubbles were introduced into the photoreactor using an air pump. An aliquot was also collected periodically with a syringe from the reactor every 15 min during a 90 min time interval and then filtered with a millipore of 0.45 μm into a cuvette. Changes in the concentration of MB were observed from its characteristic absorption at 663 nm using a UV-Vis spectrometer. Thus degradation efficiency was obtained from Eq 1[22]

$$R=\frac{Co-C}{Co}\times 100\%$$ (1)

Where *Co* is the initial MB concentration at reaction time *0* and *C* is the concentration at reaction time *t*. However, the reaction rate was also calculated using the plots of *Ln* (*Co* / *C*) against irradiation time *t* [23].

Ln(Co/C)=Kt (2)

Where the slope (*K*) is the apparent first order rate constant of the reaction and *t* is illumination time.

## Results and Discussion

### 1. XRD analysis

**Fig 1:**
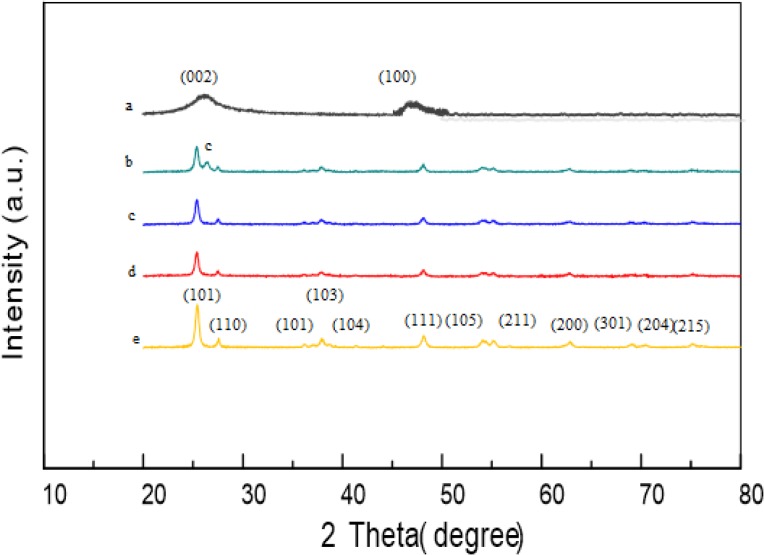
XRD patterns of acid treated MWCNTs (a), MWCNTs/TiO_2_ (b) (1:1), (c) (1:2), (d)(1:3), and (e) TiO_2_-p25.

Fig 1 presents the XRD patterns of the TiO_2_ nanopowder, acid treated MWCNTs and MWCNTs/TiO_2_ nanocomposites with different mass ratios. It was observed that the composites showed similar characteristic peaks with TiO_2_-p25 but with lower intensities (Wang et al., 2009b). Fig 1(A) depicts the XRD pattern of the MWCNT which shows characteristic peaks centered at 2θ = 26.3° and 2θ = 46.5° that may be assigned to (002) and (100) reflection of graphite, respectively. The (002) peak of the MWCNT was found to appear in the (1:1) nanocomposite (Fig 1B) denoted as **C** due to the high loading of the MWCNTs [24]. Notably, the remaining two composites do not show the presence of the typical diffraction peak of the MWCNTs. This may be attributed to the interference deriving from the main peak of anatase TiO_2_ at 25.3° that might have shielded the main MWCNT’s peak at 26.3° due to a high TiO_2_ loading [25].

It is, however, observed that the pure TiO_2_-P25 and the MWCNT/TiO_2_ composites show major peaks at 25.3, 37.0, 37.8, 53.9, 55.1, 62.7, and 75.1 corresponding to the diffractions of (101), (103), (004), (105), (211), (204) and (215) reflactions of anatase TiO_2_ teteragonal crystal planes.Whereas, the crystalline phase presenting the rutile phases of (110), (101), (111),(200) and (301) at peaks of 27.4, 36.1, 41.2, 53.2, and 69.0 indicate that the deposited TiO_2_ on the fMWCNTs existed in the anatase and rutile mixture state. Higher crystallinity favours photodegradation efficiency; thus, MWCNTs/TiO_2_ (1:3) with higher TiO_2_ content may be more crystalline hence, more active than the remaining samples.

### 2. FTIR spectra analysis

**Fig 2:**
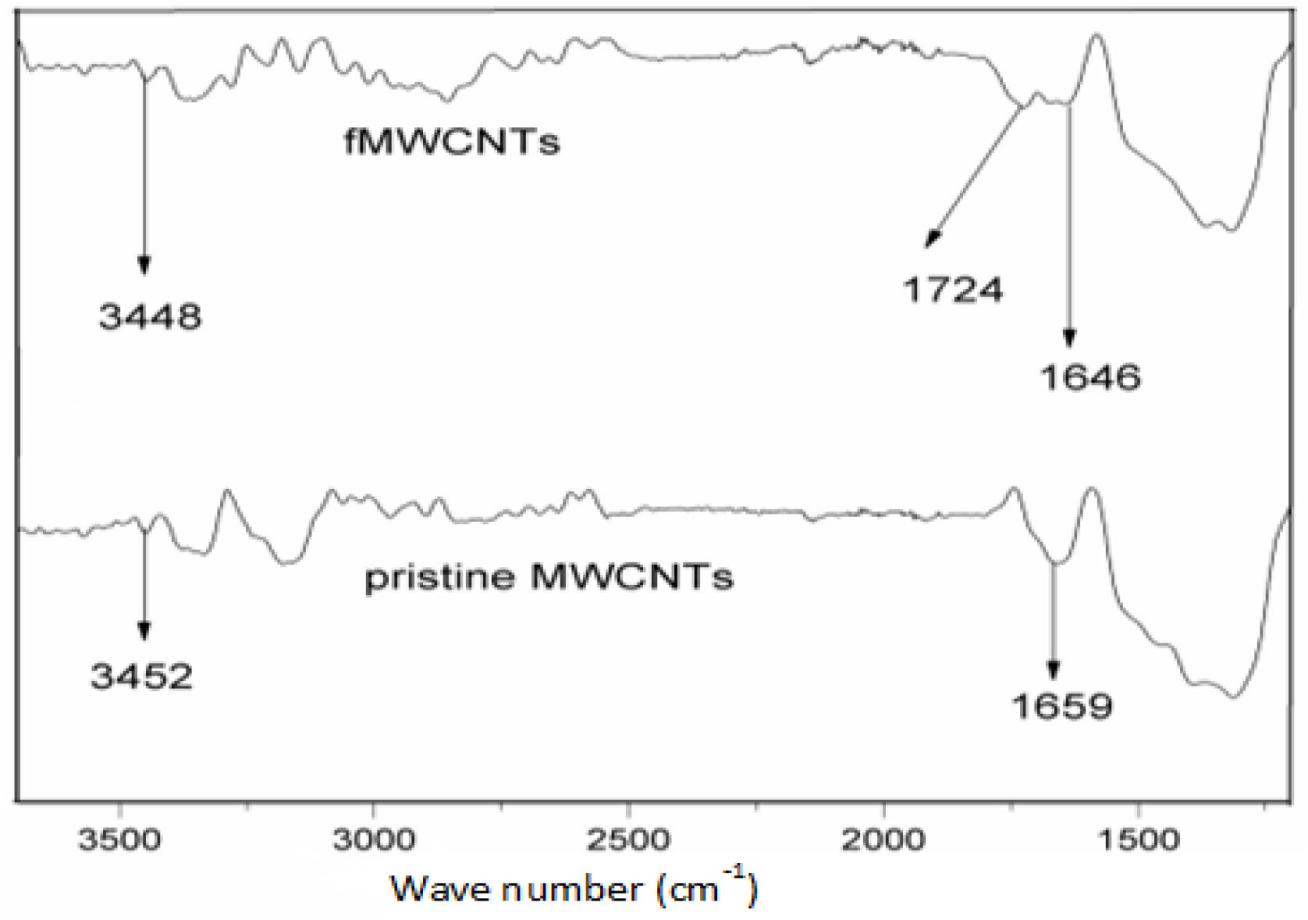
FTIR spectra of the pristine MWCNTs and functionalised MWCNTs.

The FTIR spectra of acid treated MWNCTs and pristine MWCNTs are compared in **Fig 2**. The spectra of the pristine MWCNTs showed peaks at 3452 and 1659 cm^-1^, which corresponded to hydroxyl and carbonyl functional groups, respectively [26, 27], even before the oxidation process, which may represent defects on the tubes’ surfaces. The shifts in these characteristic wave numbers in the direction of lower wave numbers 3448 and 1646 cm^-1^, respectively, indicated the presence of strong hydrogen bonds between the –OH groups and also between the –C = O– groups [28]. Whilst, the appearance of a peak at 1724 cm^-1^ in the fMWCNTs clearly indicates the formation of carboxyl functional groups (–COOH) [16]. These functional groups assured the adsorption of the metal or semiconductor nanoparticles on the surface of the MWCNTs via a hydrogen bond and Van der Waals forces. This claim was ascertained by the TEM images where the TiO_2_ nanoparticles are seen to be immobilised on the sidewalls of the MWCNTs.

### 3. TEM images

**Fig 3:**
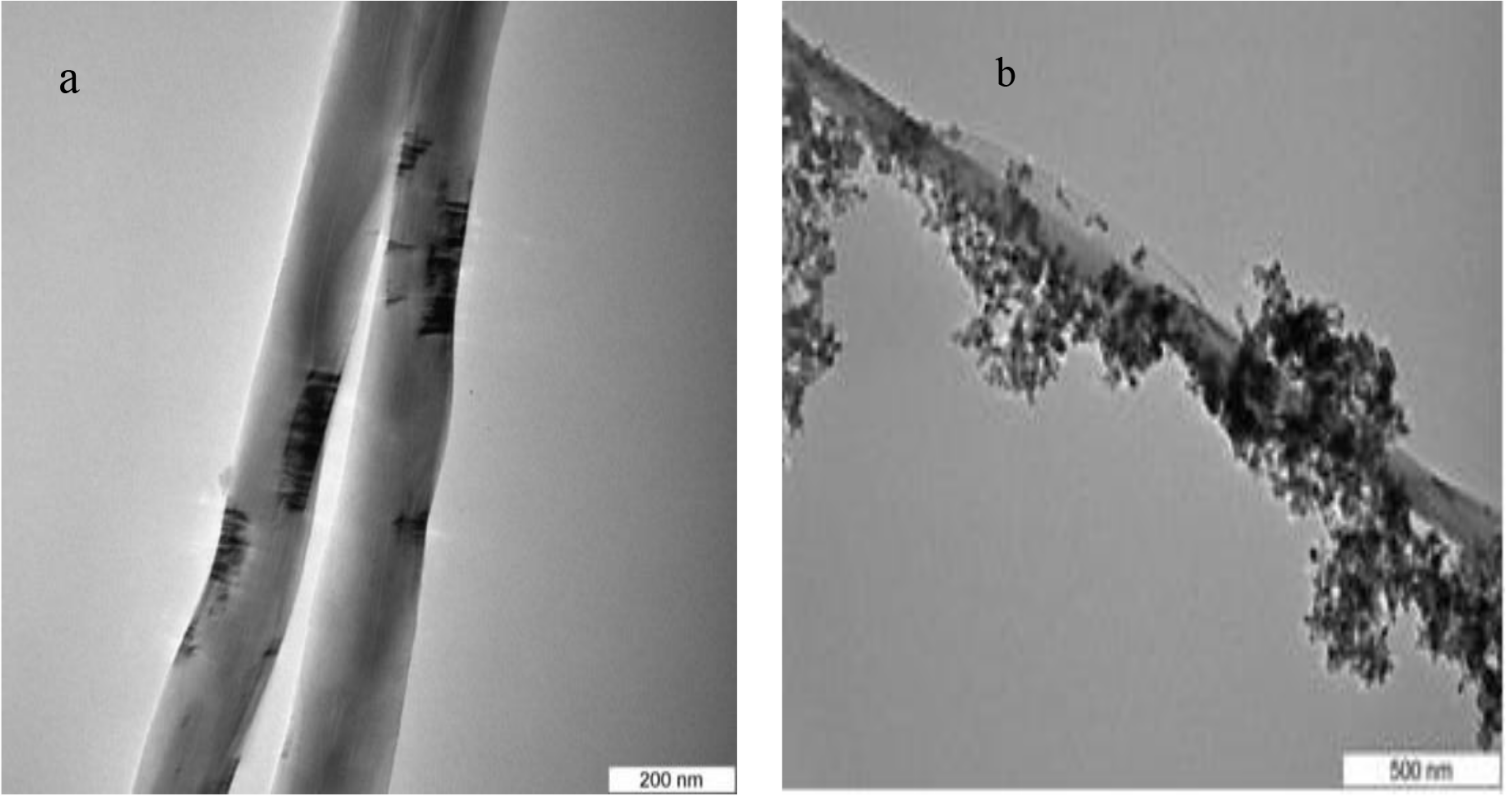
TEM images of (a) Pristine MWCNTs & (b) MWCNTs/TiO_2_ nanocomposite.

The morphologies of the pristine MWCNT and MWCNT/ TiO_2_ composites were revealed by the TEM investigation and the typical TEM images are shown in **Fig 3. Fig 3A,** is a high magnification TEM micrograph of the pristine MWCNTs; whilst, Fig 3B is the MWCNT/ TiO_2_ nanocomposite which shows no apparent aggregation of the MWCNTs. It is also observable that the composites mainly consist of spherical TiO_2_ nanoparticles and have a high tendency to agglomerate. The aggregated phenomena may be due to the high surface energy of the nanoparticles. The TEM shows average particle sizes between 27–36 nm attached on the sidewall of the MWCNTs, which is consistent with the nucleation sites observed on the oxidised MWCNTs’ surfaces.

### 4. Optical property analysis

**Fig 4:**
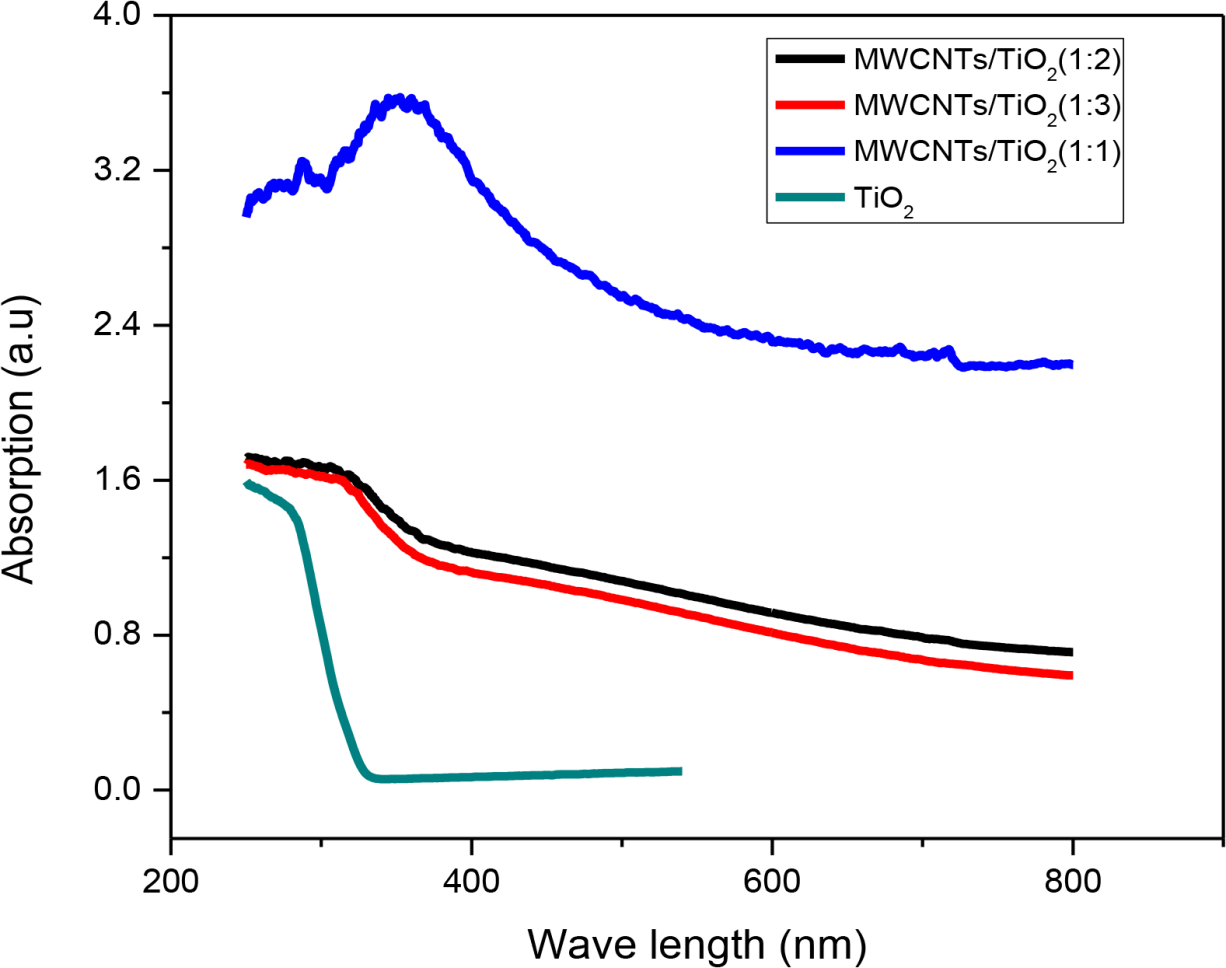
UV-vis diffuse reflectance spectra of the MWCNTs/TiO_2_ nanocomposites of different weight ratios and TiO_2_- P25.

The optical properties of the prepared MWCNT/ TiO_2_ nanocomposites have been determined by UV-vis spectroscopy, and presented in **Fig 4**. The figure confirms that the MWCNT/TiO_2_ composites can absorb significantly more light in the 420–800 nm regions as compared to the commercial TiO_2_-P25. The presence of MWCNT leads to the extension of the TiO_2_ absorption threshold to the visible region. However, this collaborates with some previous studies that the absorption in the visible range increases with increasing the MWCNT dose [15].

### 5. Photoluminescence (PL) spectra

**Fig 5:**
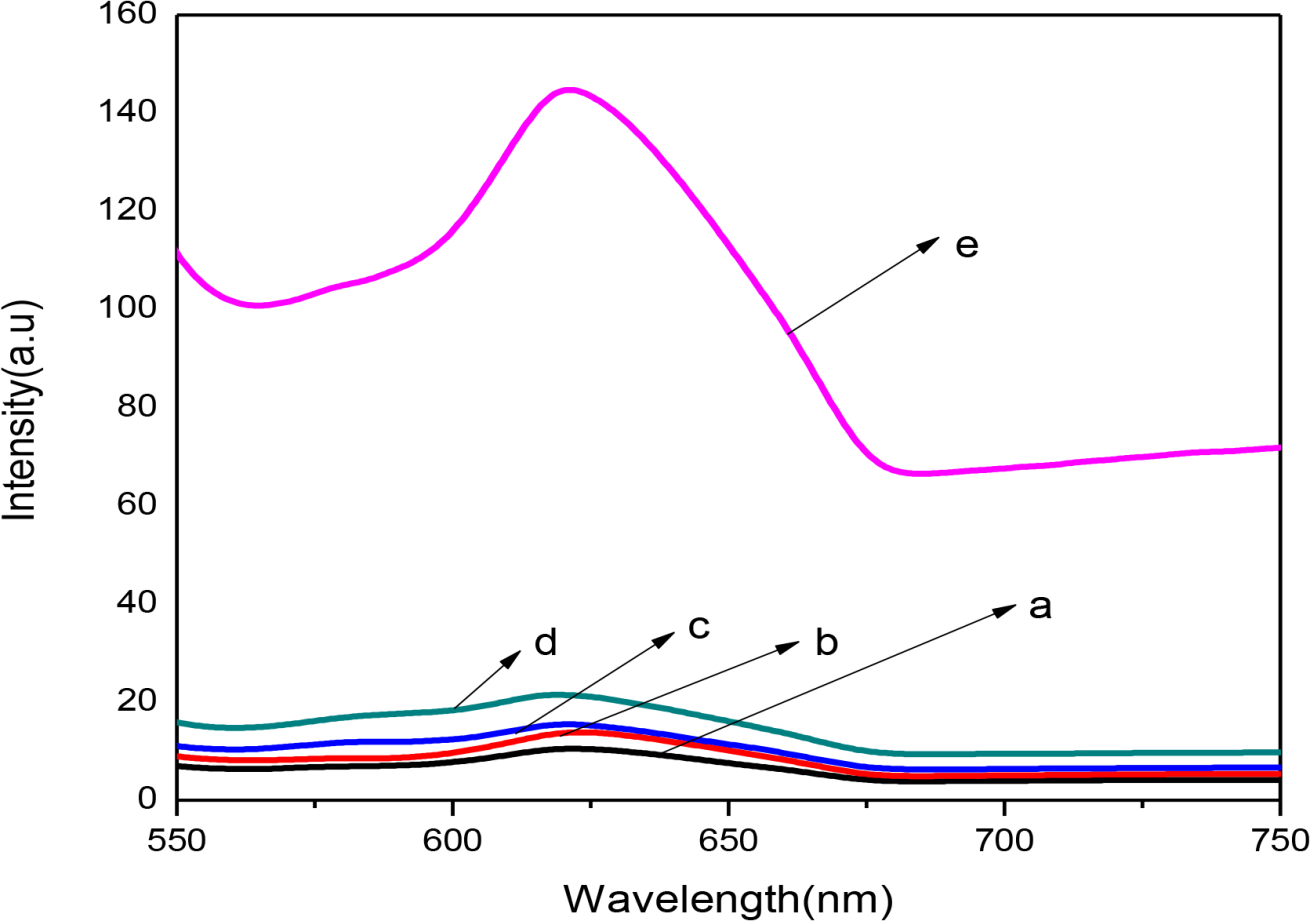
PL spectrum of MWCNTs (a), MWCNTs/TiO_2_ nanocomposites (b)(1:1), (c)(1:2), (d)(1:3) and (e)TiO_2_-p25.

Apart from the wide band gap phenomenon, the recombination of photogenerated holes and electrons is another drawback affecting the photocatalytic activity of the TiO_2_ semiconductor. The PL emission spectrum has been famously used in investigating the efficiency of charge carrier trapping, immigration and transfer. It is also applicable in understanding the fortune of e^-^/h^+^ pairs in semiconductor particles [29]. During the photocatalysis, photons are emitted leading to photoluminescence as a result of electron /hole pair recombination. This attribute may be due to the reverse radiative deactivation from the excited-state of the Ti species. The influence of the MWCNTs on the recombination of e^-^/h^+^ produced by titanium oxide is shown in **Fig 5**. On comparison, it is obvious that the TiO_2_ powder showed a broad PL emission band. And, the peak intensity of the TiO_2_ was higher than that of the nanocomposites and decreased as the MWCNTs’ loading increased. This suggested that the formation of MWCNT and TiO_2_ nanocomposites can reduce radiative e^-^/h^+^ pair recombination.

### 6. Effect of MWCNTs/TiO_2_ mass ratios

**Fig 6:**
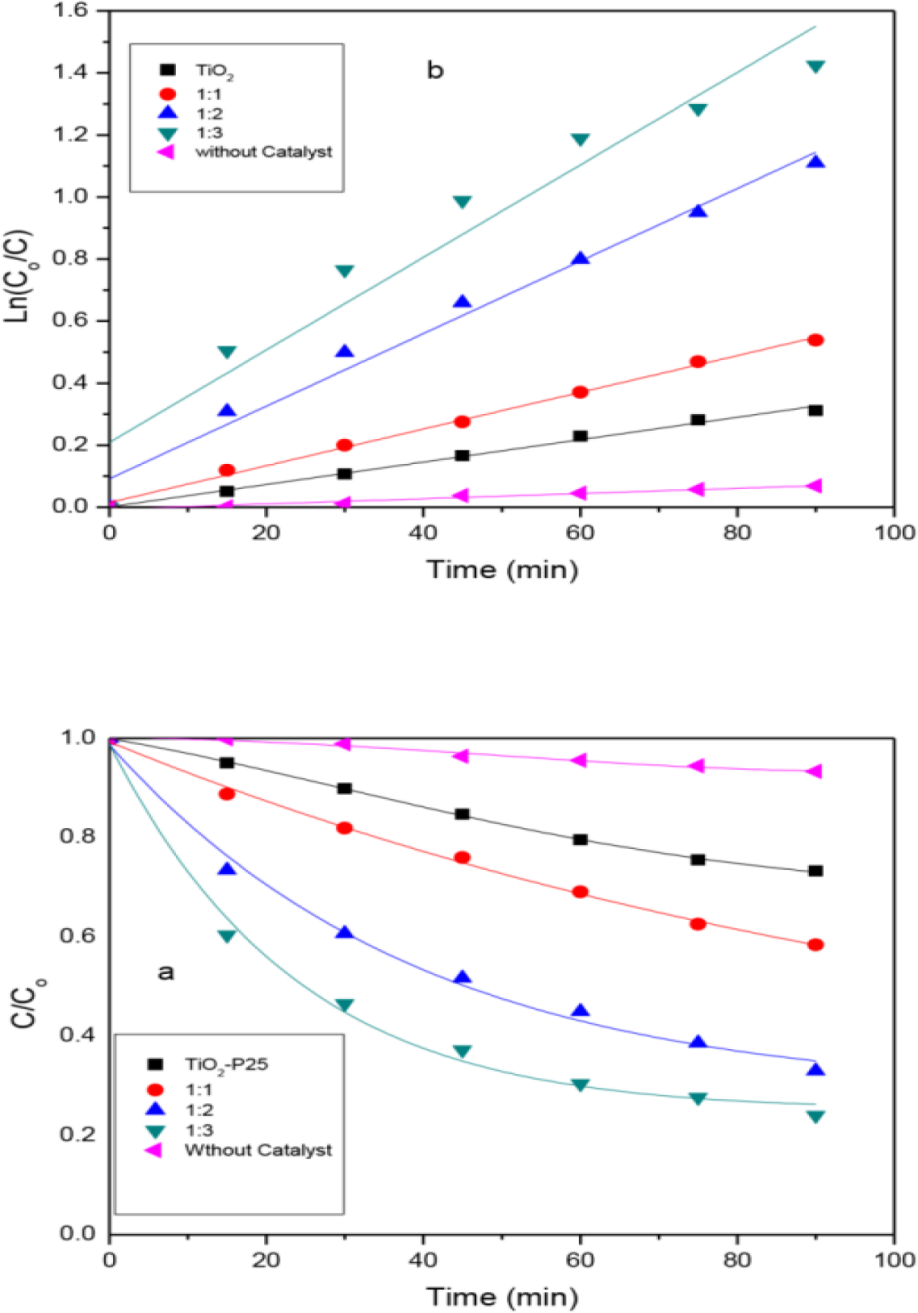
A normalised concentration (*C*/*C*_0_); *C*_0_ initial concentration at *t = 0*, *C* concentration at time *t* versus reaction time (*t*) for different samples tested(a); and the kinetic plots of apparent first-order linear transform ln (*C*_*o*_ */C*) against time of irradiation *(t)* (b).

**Fig 6.** presents the photodegradation of 10 ppm MB by TiO_2_-P25, a blank solution and MWCNTs/TiO_2_ nanocomposites under visible light irradiation. The reaction was carried out under the following experimental condition: 10 ppm, 500 ml of MB dye, temperature of 25°C, pH 7 and 0.1 g weight of the catalysts. The results indicate that no significant degradation of MB occurs in the absence of any catalysts for 90 min of visible light irradiation. The rate constant was the lowest without a catalyst (8.3898 x 10^–4^ min^-1^) and the removal efficiency was only 5.54%. The TiO_2_-P25 had a slower rate in C/C_o_ and also a lower first order rate constant (*K*), with respect to the remaining samples; thus, it had poor activity under visible light due to its wide band gap. Similarly, the poor activity of the TiO_2_-P25 may be attributed to the absence of MWCNTs; as such, the TiO_2_ nanoparticles were bound to the agglomeration due to their high surface energy thereby decreasing the surface area leading to the poor adsorption of the MB molecules. In addition, the photocatalytic activity increased in the following order: TiO_2_, (1:1), (1:2) and (1:3), which is in agreement with the band gap energy of the samples.

The photodegradation of organic dye follows the first-order reaction having simplified Langmuir-Hinshelwood model when the initial concentration was too small [23]. The order of the reaction with respect to methylene blue was calculated from (Fig 6B), i.e., the graph of ln(*C*o/*C*) against reaction time t according to (Eq 2), where ***k*** represented the first-order rate constant. And the removal efficiency (R) was obtained from (Eq 1) and the average lowering rate (C/C_o_) with respect to the samples were shown in Table 1. Based on the kinetic plots, the (1:3) nanocomposite had the highest rate constant (149 x 10^–4^ min^-1^), a faster lowering rate (**Fig 6B**) and a higher degradation rate of 75.96% at 90min irradiation time as compared to the 1:2 (117 x 10^–4^ min^-1^ and 67.02%) and 1:1(59 x 10^–4^ min^-1^ and 41.63%) photocatalysts. Obviously, both (1:3) and (1:2) have *K* values that doubled that of (1:1) and tripled that of the TiO_2_-p25. The increase in the TiO_2_ content in the composites seems to be because of a decrease in the MWCNTs. Hence, a small amount of MWCNTs acted as a “dispersing template or support” that controlled the morphology of the TiO_2_ nanoparticles in the MWCNT/TiO_2_ nanocomposite, preventing agglomeration and serving as a reservoir of electrons to trap electrons and prolong the lifetime of the e/h pairs. Therefore, these roles of the MWCNTs in tailoring the morphological structure of the MWCNT/TiO_2_ composites have been reported to enhance the photocatalytic activity of the functional material [30, 31]. It was, however, reported that at higher TiO_2_ mass ratios above optimum, the catalytic activity of a catalyst may decrease [15]. Thus, it is established that a suitable dose of TiO_2_ is crucial for the optimum synergy between the MWCNTs and the TiO_2_ nanocomposites.

**Table 1:** The apparent rate constant (*k*), photodegradation efficiency (R) and average C/C_o_ at 10ppm MB initial concentration.

Catalyst	K (min^1^) (x 10^–4^)	Photodegradation efficiency (R) (%)	Average C/C_o_
**TiO_2_**	36	26.74	0.8298
**MWCNTs/TiO_2_ (1:1)**	59	41.63	0.7276
**MWCNTs/TiO_2_ (1:2)**	117	67.02	0.5041
**MWCNTs/TiO_2_ (1:3)**	149	75.96	0.3771

### 7. Effect of the MB initial concentration on the optimum photocatalyst

**Fig 7:**
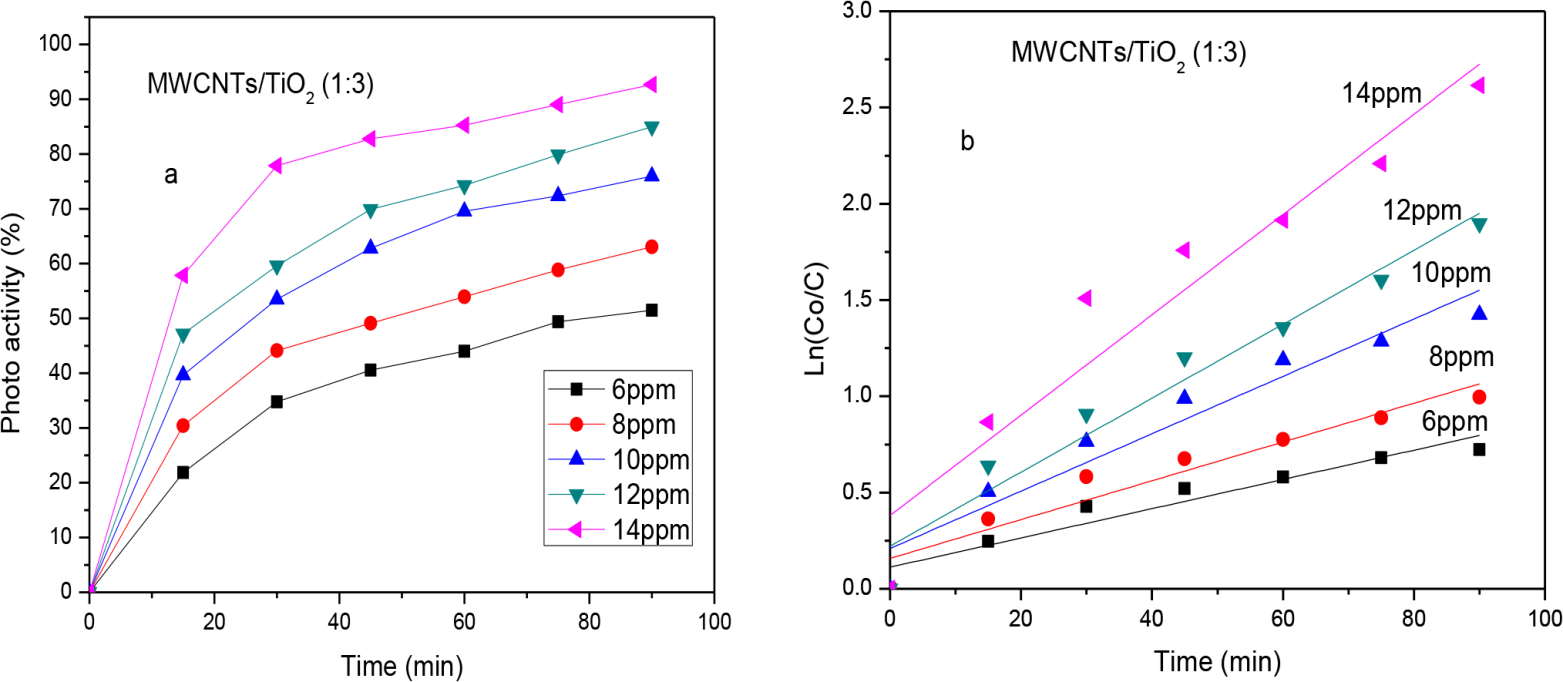
Photodegradation efficiency versus time of irradiation (a), kinetic plots of apparent first-order linear transform ln (C_o_/C) against time of irradiation (t) (b) under optimised conditions.

The effect of the MB initial concentration on the optimum photocatalyst was studied by varying the initial concentration from 6 ppm to 14 ppm whilst keeping other experimental conditions unchanged as described in section 6. As revealed in **Fig 7, a** noticeable decrease in the initial dye concentration was observed even before the start of the photocatalysis reaction; this was because of the adsorption/desorption on the catalyst substrate. It was observed that the degradation efficiency (***R***) depended on the initial dye concentration and the ***k*** value increased with an increasing dye initial concentration. The degradation efficiency (R), the apparent first order rate constant (K) and the respective adjusted R square at different MB initial concentrations were presented in Table 2. The results also indicated that the photocatalytic activity initially increased significantly from 51.47% to 92.69% with an increasing in the initial MB concentration from 6 ppm to 14 ppm and the apparent rate constant, *k*, reached the highest value of 261 × 10^−4^ min^−1^ (Fig 7B). This may be attributed to the fact that the tendency of the reactions between the MB and the free radicals produced increased when the initial concentration increased.

**Table 2:** The apparent rate constant (*k*), photodegradation efficiency (R) and adjusted R-square at different MB initial concentrations.

MB concentration (ppm)	K (min ^-1^) (x 10^–4^)	Photodegradation efficiency (R) (%)	R^2^
6	76	51.47	0.90735
**8**	101	63.06	0.90930
**10**	150	75.95	0.92191
**12**	192	84.99	0.95189
**14**	261	92.69	0.91050

Liu and co-workers reported that a higher concentration of dye above the optimum level may hinder visible light and consequently, the light cannot trigger photoelectron generation. Thus, this decreases the concentration of hydroxyl free radicals that play an important role in photocatalytic degradation [32].

### 8. Effect of pH

The pH of the MB aqueous solution under the optimised experimental condition was also investigated. The pH enhanced the dissociation and adsorption of the organic compounds, charge on the photocatalyst and other physical and chemical properties of the photodegradation system [33]. The aqueous solution was adjusted to 1.9, 7.0 and 10.3pH with 0.1M NaOH and HCl. All other experimental conditions were kept constant as described in section 6.

**Fig 8:**
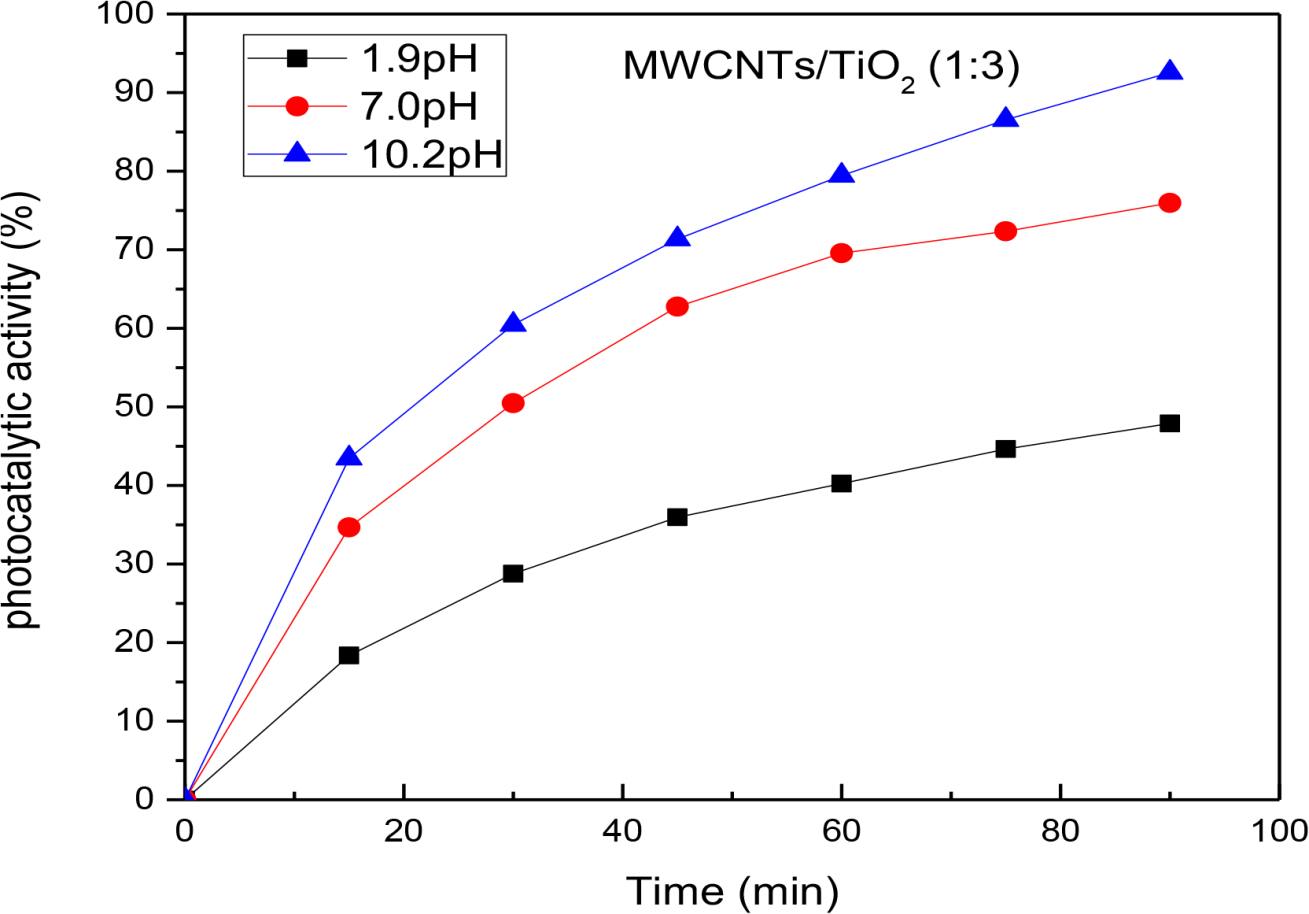
The influence of pH on the photoactivity of the MWCNTs/TiO_2_ (1:3) catalyst.

Fig 8 shows the MB photodegradation versus time. The figure shows a higher degradation rate at alkaline and neutral mediums. 50% degradation of the MB was observed at *half-life* = 20.8 and 29.3 min for the pH at 10.2 and 7.0, respectively. However, in the acidic environment, only 45% of the MB was degraded for 90 min. This may be attributed to the amphoteric nature of the TiO_2_ photocatalyst, where it reacted as an acid or base. The TiO_2_ surface behaved as a cation and an anion in acidic and basic solutions, respectively. Due to the cationic condition of methylene blue it was expected that at acidic pH values, its adsorption would be thermodynamically unfavourable. As such, mutual repulsion may have resulted between the TiO_2_ and MB molecules thus reducing the photodegradation efficiency.

## Conclusion

A simple technique was employed in the synthesis of the MWCNT/TiO_2_ nanocomposites, which showed enhanced photoactivity in the decay of the methylene blue under the influence of visible light in comparison with the titanium nanopowders (P25). Employing various techniques (FT-IR, TEM, XRD, UV-vis, PL,) to characterise the material features, we have demonstrated that the MWCNTs/TiO_2_ nanocomposite possesses unique optical and chemical properties. Their reactivity to visible light, however, is possibly explained as due to the band gap shrinking and charge-trapping effects. In addition, MWCNTs may also act as a photosensitiser starting a series of electron transfers and ultimately creating oxide radicals, causing methylene blue degradation. The magnitude of the improved photoactivity depends on the relative mass ratio of MWCNT/TiO_2_, pH of the solution and initial dye concentration.
